# Differential Detection of Human Papillomavirus Genotypes and Cervical Intraepithelial Neoplasia by Four Commercial Assays

**DOI:** 10.1128/JCM.01321-16

**Published:** 2016-10-24

**Authors:** Matejka Rebolj, Jesper Bonde, Sarah Preisler, Ditte Ejegod, Carsten Rygaard, Elsebeth Lynge

**Affiliations:** a Department of Public Health, University of Copenhagen, Copenhagen, Denmark; b Clinical Research Centre, Copenhagen University Hospital Hvidovre, Hvidovre, Denmark; c Department of Pathology, Copenhagen University Hospital Hvidovre, Hvidovre, Denmark; University of Texas Medical Branch

## Abstract

Laboratories now can choose from >100 human papillomavirus (HPV) assays for cervical screening. Our previous analysis based on the data from the Danish Horizon study, however, showed that four widely used assays, Hybrid Capture 2 (HC2), cobas, CLART, and Aptima, frequently do not detect the same HPV infections. Here, we determined the characteristics of the concordant samples (all four assays returning a positive HPV test result) and discordant samples (all other HPV-positive samples) in primary cervical screening at 30 to 65 years of age (*n* = 2,859) and in a concurrent referral population from the same catchment area (*n* = 885). HPV testing followed the manufacturers' protocols. Women with abnormal cytology were managed according to the routine recommendations. Cytology-normal/HPV-positive women were invited for repeated testing in 18 months. Screening history and histologically confirmed cervical intraepithelial neoplasia (CIN) in 2.5 years after the baseline testing were determined from the national pathology register. HPV-positive women undergoing primary screening having concordant samples were more likely to harbor high-risk infections and less likely to harbor only low-risk infections than women with discordant samples. Additionally, assay signal strengths were substantially higher in concordant samples. More than 80% of ≥CIN2 results were found for women with concordant samples, and no ≥CIN2 results were found when the infection was detected by only one assay. These patterns were similar in the referral population despite the younger age and higher number of HPV infections. HPV test result discordance identified a cluster of low-risk HPV infections that were hardly ever associated with high-grade CIN and, almost exclusively, represented false-positive screening findings.

## INTRODUCTION

Human papillomavirus (HPV) assays will be used for cervical cancer screening (to select women with a high risk of high-grade cervical intraepithelial neoplasia [CIN], termed ≥CIN2) and for monitoring of the effectiveness of HPV vaccination (to determine the changes in infection epidemiology). Currently, laboratories can choose from >100 commercially available HPV assays (1). These assays utilize different technologies to detect HPV infections (2). The randomized controlled trials of primary screening studied only two assays, Hybrid Capture 2 (HC2) and GP5+/6+ PCR (3). Therefore, it has been recommended that other assays should be compared to them in terms of the detection of HPV infections and CIN (4).

In the Danish split-sample Horizon study comparing HC2 (Qiagen, Gaithersburg, MD), cobas (Roche, Pleasanton, CA), CLART (Genomica, Madrid, Spain), and Aptima (Hologic, San Diego, CA) HPV assays, the detection of HPV infections depended on the assay. This was particularly pronounced in women undergoing primary cervical screening at ≥30 years, where only 29% of all positive test results were concordant on all four compared assays (5). The remaining 71% (partially) discordant positive test results reflected infections in 16% of all screened women tested with the four assays. For comparison, only 4% of all screened women had abnormal cytology, pointing out that HPV assay discordance is not of trivial magnitude. The same pattern was seen in studies undertaken in populations with substantially different HPV prevalence rates and using other sample storage media (5).

This unexpected finding warrants an additional in-depth analysis, as it may have implications for the two intended uses of HPV assays. Hence, our aim here was to study an infected woman's likelihood of harboring high-risk HPV and high-grade CIN depending on the degree of the discordance between the HPV test results. To gain insight into the patterns across various populations, we studied two groups of women: those undergoing primary screening and those with (recent) abnormalities (i.e., a referral population).

## MATERIALS AND METHODS

### Study design.

The study design was described in detail previously (5–11). In short, consecutive routine SurePath samples from 5,034 women arriving at the Department of Pathology of Copenhagen University Hospital Hvidovre in June to August 2011 were collected and tested with liquid-based cytology (LBC; reported using the Bethesda 2001 system) and the four HPV assays (this constituted the baseline testing). By linkage to the Danish National Pathology Register (Patobank) (12) from 1 January 2000 onwards, the samples were categorized as primary (screening) samples or as samples taken for follow-up of recent abnormalities. Screening samples were defined as those without a previous histological diagnosis of cervical cancer, histologically confirmed CIN in ≤3 years, atypical squamous cells of undetermined significance (ASCUS) or non-CIN cervical histology in ≤15 months, or a more severe cytological abnormality, inadequate cytology, or positive HPV test in ≤12 months. Reflecting routine practice, these samples included a small proportion of samples taken for investigation of symptoms. All other samples were follow-up samples.

Women with abnormal cytology were monitored according to the routine guidelines in use for the laboratory's catchment area (colposcopy was performed if the women had high-grade squamous intraepithelial neoplasia [HSIL] or worse, atypical glandular cells [AGC], atypical squamous cells not excluding HSIL [ASC-H], adenocarcinoma *in situ* [AIS], HC2-positive ASCUS at ≥30 years, abnormal repeated testing following ASCUS at <30 years, low-grade squamous intraepithelial lesions [LSIL], or HC2-negative ASCUS at ≥30 years). Women with normal cytology and a positive test result on at least one HPV assay were invited, according to the study protocol, for repeated cytology and HPV testing in 18 months after the baseline. Follow-up samples with abnormal cytology or a positive HC2 test result elicited a referral for colposcopy. All colposcopies were undertaken under routine conditions either by a hospital or privately practicing gynecologists. In Denmark, it is recommended that directed biopsy specimens be taken from all suspicious areas after application of acetic acid and random biopsy specimens from four quadrants if lesions are not visible. The Hvidovre laboratory evaluated almost 90% of the high-grade CIN biopsy specimens included in the study. During the study period, between four and seven pathologists evaluated cervical cytology and gynecological histology in this laboratory. Most samples were read by one pathologist. p16 staining was not used systematically. The remaining approximately 10% of high-grade CIN biopsy specimens were evaluated in other Danish hospitals or by private pathologists. Data on the most severe histological diagnosis during follow-up were retrieved from the national Patobank in December 2013, covering the period of about 2.5 years after baseline. Follow-up was highly complete for women with abnormal baseline cytology (ca. 95%) and moderately complete (ca. 60%) for women with cytology-normal/HPV-positive test results (6).

### HPV testing.

All assay testing and sample storage were undertaken in strict concordance with the protocols agreed upon by all manufacturers prior to the study (see the supplemental material). The instrumentation and software were used as supplied and maintained by the manufacturers. HC2 testing was undertaken on the postquot LBC material. cobas, CLART, and Aptima testing was undertaken on the original residual material, diluted approximately 1:1 in SurePath. HC2 detects 13 high-risk HPV genotypes collectively. The assay is based on hybridization of HPV DNA to a high-risk HPV RNA probe cocktail. cobas is a real-time PCR analysis detecting the 13 high-risk HPV genotypes and HPV 66. The assay separately identifies HPV 16 and HPV 18, while the remaining 12 genotypes are detected collectively. The CLART assay is a PCR-based low-density microarray detecting 35 defined genotypes, including the 13 high-risk genotypes. All genotypes are reported individually. Aptima detects E6/E7 mRNA expression of the 13 high-risk HPV types and HPV 66 collectively. HC2, cobas, and Aptima are FDA-approved assays and have also been validated according to the protocol defined by the international assay validation guidelines (2, 4).

### Statistical analysis.

The primary screening population was defined as women with screening samples at 30 to 65 years of age without invalid HPV test results (*n* = 2,859, 56.8% of all 5,034 included women; 10 were excluded because of invalid HPV test results). The referral population included women with follow-up samples and women with screening samples showing abnormal cytology at any age, excluding women with invalid HPV test results (*n* = 885, 17.6%).

HC2 was positive when the relative light units per cutoff (RLU/CO) value was ≥1. cobas was positive when channels 16, 18, and/or other high-risk genotypes had critical threshold (*C_T_*) values of ≤40.5, ≤40.0, and ≤40.0, respectively (for samples where >1 channel returned a positive test result, we considered the channel with the strongest signal, i.e., the lowest *C_T_* value). Aptima was considered positive when the signal-to-cutoff (S/CO) value was ≥0.5. CLART was considered positive when ≥1 high-risk HPV genotype was detected. In line with the classification by the International Agency for Research on Cancer, genotypes 16, 18, 31, 33, 35, 39, 45, 51, 52, 56, 58, 59, and 68 were considered high risk (13).

We categorized samples by the number of positive HPV test results (one through four). For each category, we determined the proportions of samples with HPV 16 infections, with infections with the other 12 high-risk genotypes (excluding HPV 16), with only low-risk HPV infections, and without a single detected HPV genotype (from among 35 detectable by CLART); these proportions were mutually exclusive. Separately, we determined the proportions of high-risk samples with multiple infections (defined as detection of ≥2 genotypes with or without low-risk genotypes) and with abnormal cytology (≥ASCUS). Genotypes were reported as detected by CLART. Trends in the proportions of genotype detection by category were tested with the Mantel-Haenszel χ^2^ test for trend. We determined the median signal strengths and the associated interquartile ranges (IQR) on the HC2, cobas, and Aptima assays for samples that tested positive on only one assay compared to samples that tested positive on all four assays. The 95% confidence intervals (CI) for relative proportions (RP) comparing screening and referral populations were calculated assuming lognormal distribution. Analyses were undertaken using IBM SPSS Statistics, version 19.

### Ethics statement.

The baseline testing on the residual material was undertaken as a quality development study and did not require ethical approval, in line with Danish regulations of biomedical research. The Ethical Committee of the Danish Capital Region approved the follow-up of HPV-positive/cytology-normal women (H-4-2012-120), and women signed informed consent. The Danish Data Inspection Agency was notified of the study (notification numbers 2010-41-5594 and AHH-2015-080/I-Suite: 04109).

## RESULTS

### Screening population.

**FIG 1:**
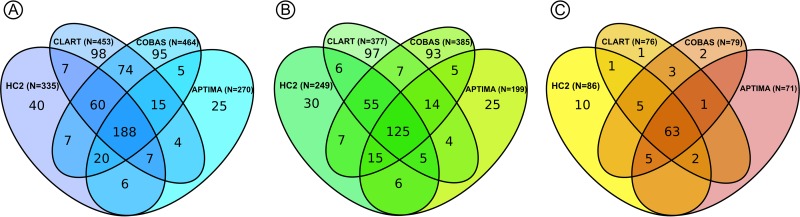
Distribution of test results at baseline in HPV-positive women with screening samples at age 30 to 65 years. (A) All screened women (*n* = 651). (B) Women with normal cytology (*n* = 558). (C) Women with abnormal cytology (*n* = 93).

The mean age of the 2,859 women was 42.7 years (range, 30 to 65 years; standard deviations [SD], 9.6). Of these, 651 (22.8%) had at least one positive HPV test result at baseline (Table 1). Only 188 (28.9%) of the 651 HPV-positive women tested positive on all four assays. Samples with only one positive HPV test result represented ca. 10 to 20% of all positive test results on each assay (e.g., 40/355 [11.3%] for HC2 and 98/453 [21.6%] for cobas) (Fig. 1), and combined they accounted for 258 (39.6%) of all positive test results in the study. In 558 women with positive HPV test results and normal cytology, 125 women (22.4%) tested positive on all four assays. In 93 women with positive HPV test results and abnormal cytology, all four assays tested positive in 63 women (67.7%).

**TABLE 1:** Description of HPV infections in samples with one, two, three, or all four HPV assays returning a positive result

HPV test result	Total no. (column %)	No. (row %) with abnormal cytology	No. (row %) of multiple infections	HPV genotype distribution^*a*^ (no. [row %])			
				HPV 16	Non-HPV 16 high-risk HPV	Only low-risk HPV infection	No HPV genotype
Primary screening at 30–65 yr (*n* = 2,859)^*c*^							
1 positive	258 (39.6)	13 (5.0)	37 (14.3)	16 (6.2)	82 (31.8)	52 (20.2)	108 (41.9)
2 positives	103 (15.8)	4 (3.9)	35 (34.0)	16 (15.5)	69 (67.0)	9 (8.7)	9 (8.7)
3 positives	102 (15.7)	13 (12.7)	57 (55.9)	26 (25.5)	56 (54.9)	11 (10.8)	9 (8.8)
4 positives	188 (28.9)	63 (33.5)	121 (64.4)	41 (21.8)	147 (78.2)	0 (0)	0 (0)
Total positives	651 (100)	93 (14.3)	250 (38.4)	99 (15.2)	354 (54.4)	72 (11.1)	126 (19.4)
*P*^*b*^		<0.001	<0.001	<0.001	<0.001	<0.001	<0.001
Referral population (*n* = 885)^*c*^							
1 positive	89 (17.1)	31 (34.8)	15 (16.9)	4 (4.5)	16 (18.0)	37 (41.6)	32 (36.0)
2 positives	63 (12.1)	15 (23.8)	30 (47.6)	14 (22.2)	33 (52.4)	13 (20.6)	3 (4.8)
3 positives	77 (14.8)	38 (49.4)	37 (48.1)	13 (16.9)	50 (64.9)	12 (15.6)	2 (2.6)
4 positives	290 (55.9)	233 (80.3)	204 (70.3)	100 (34.5)	190 (65.5)	0 (0)	0 (0)
Total positives	519 (100)	317 (61.1)	286 (55.1)	131 (25.2)	289 (55.7)	62 (11.9)	37 (7.1)
*P*^*b*^		<0.001	<0.001	<0.001	<0.001	<0.001	<0.001

a As detected by the CLART assay.

b Determined by the Mantel-Haenszel Χ^2^ test for trend.

c Primary screening indicates women with a cytological sample at age 30 to 65 years with no recent cervical abnormalities (see Materials and Methods). Referral population indicates women with an abnormal cytological sample attending primary screening or women attending follow-up for recent cervical abnormalities at any age (see Materials and Methods).

Women with a positive test result on only one HPV assay were less likely to have HPV 16 infections, infections with other high-risk genotypes, multiple HPV infections, and abnormal cytology than women with a positive test result on >1 HPV assay (*P* < 0.001). Their samples were also more likely to contain only low-risk or no HPV genotype (*P* < 0.001). As this comparison could have been biased by the double role of the CLART assay (i.e., being one of the compared assays at the same time as having the role of adjudicating HPV genotypes), we additionally studied the concordance comparing only the HC2, cobas, and Aptima assays, with CLART used exclusively for an adjudication of the genotypes present in the sample. This comparison revealed essentially the same patterns (see Table S1 in the supplemental material).

Signal strength values indicated that samples with only one positive HPV test result were relatively weakly positive compared to the samples from women with four positive test results, and their medians were close to the manufacturer-recommended threshold cutoffs for positive test results (Table 2). The median signals in samples with all four assays returning a positive test result were, on the other hand, substantially different from the manufacturers' threshold values. The IQRs for the weakly and strongly positive categories of samples did not overlap.

**TABLE 2:** Median signal strengths for detected HPV infections and CIN lesions in samples with one or all four HPV assays returning a positive result

No. of positive HPV test results	Median (IQR) signal strength for detected HPV infections			No. of detected histologically confirmed CIN lesions^*a*^ (% of total)		
	HC2	cobas	Aptima	≤CIN1^*b*^	≥CIN2	≥CIN3
Primary screening at 30–65 yr						
1	2.3 (1.5–8.5)	39.2 (38.3–39.8)	1.9 (0.9–6.5)	33/100 (33.0)	0/50 (0)	0/38 (0)
4	53.9 (11.5–200.2)	28.2 (25.7–31.3)	10.8 (8.4–11.8)	34/100 (34.0)	42/50 (84.0)	31/38 (81.6)
Referral population						
1	5.2 (1.7–24.4)	38.9 (37.7–39.3)	2.1 (0.6–5.5)	35/176 (19.9)	3/129 (2.3)	3/89 (3.4)
4	147.9 (32.8–512.1)	26.8 (23.8–29.6)	11.1 (9.8–14.8)	95/176 (54.0)	105/129 (81.4)	72/89 (80.9)

a Among lesions detected by at least one HPV assay. One woman with all four HPV assays returning negative test results had CIN3. Recalculated as detection per 100 women undergoing HPV testing, the proportions of ≤CIN1 were 3.5% (100/2,859) in the screening population and 19.9% (176/885) in the referral population. The proportions of ≥CIN2 were 1.7% (50/2,859) and 14.6% (129/885), respectively, and the proportions of ≥CIN3 were 1.3% (38/2,859) and 10.1% (89/885), respectively.

b Includes normal histology and histologically confirmed CIN1.

**FIG 2:**
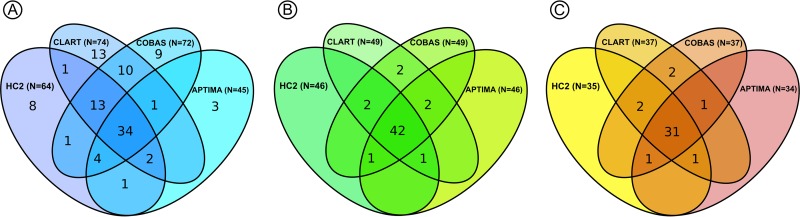
Distribution of histologically confirmed CIN lesions in HPV-positive women with screening samples at age 30 to 65 years, with follow-up from the baseline in June-August 2011 to December 2013. Linkage was performed through the national Patobank. (A) Women with ≤CIN1 (*n* = 100). (B) Women with ≥CIN2 (*n* = 50). (C) Women with ≥CIN3 (*n* = 38).

Among the 2,859 women, HPV assays detected 100 (3.5%) histologically confirmed ≤CIN1, 12 (0.4%) CIN2, 50 (1.7%) ≥CIN2, and 38 (1.3%) ≥CIN3 cases (Table 2). All four assays jointly returned a positive test result in 34 (34.0%) of the 100 ≤CIN1 cases. This was 42 out of 50 women (84.0%) with ≥CIN2 and 31 out of 38 women (81.6%) with ≥CIN3 (Fig. 2). Among women who tested positive on only one HPV assay, none had ≥CIN2, although low-grade CIN was not infrequent.

### Referral population.

The mean age of the 885 women was 34.0 years (range, 18 to 89 years; SD, 10.8 years), and 519 (58.6%) had HPV infections detected on one or more assays. Of the 519 samples with at least one positive HPV test result, 89 (17.1%) were positive on only one, and 290 (55.9%) were positive on all four assays.

These HPV-positive women differed from those in the screening population. They were more likely than the screening population to be infected with HPV 16 (RP, 1.66; 95% CI, 1.31 to 2.10; calculated as 131/519 versus 99/651) (Table 1) and have multiple HPV infections (RP, 1.43; 95% CI, 1.27 to 1.63; calculated as 286/519 versus 250/651). These women were less likely to have no genotypes detected in their samples (RP, 0.37; 95% CI, 0.26 to 0.52; calculated as 37/519 versus 126/651). In this comparatively young and high-risk population, the median assay signal strengths were stronger and the concordance in detecting infections between the four HPV assays was better than that in the screening population. Nevertheless, also here the median assay signals were stronger for women with concordant HPV test results than for women with discordant HPV test results (Table 2).

Among the 885 women, HPV assays detected 176 (19.9%) women with histologically confirmed ≤CIN1, 40 (4.5%) with CIN2, 129 (14.6%) with ≥CIN2, and 89 (10.1%) with ≥CIN3. Among women with high-grade CIN, the concordance between the four HPV assays was similar to that in the screening population. Of the 129 women with ≥CIN2, 105 (81.3%) were positive on all four assays, as were 72 (80.9%) of the 89 women with ≥CIN3. High-grade CIN was only rarely found in women with only one positive HPV test result (2.3% of 129 women with ≥CIN2 and 3.4% of 89 women with ≥CIN3).

## DISCUSSION

### Main findings.

Screening samples in which the HC2, cobas, CLART, and Aptima assays showed HPV status discordance represented a cluster with a lower risk of ≥CIN2 than samples in which all four assays detected HPV infections. Discordant samples had weaker assay signals, were less likely to contain high-risk genotypes, multiple infections, and high-grade CIN, and more likely to harbor only low-risk or no HPV genotype. These differences between weakly and strongly positive samples were also found in the referral population with a lower average age and a higher prevalence of high-risk infections, suggesting that our findings can be generalized across various populations.

### Strengths and weaknesses of the study.

The study was undertaken on fresh routine samples. All samples were handled in one laboratory by the same staff. By having access to the women's screening histories, we could determine which samples were taken for screening purposes, even though this information was not registered routinely. This is one of a few studies that compared several HPV assays on primary screening samples with active follow-up of women with positive HPV test results and normal cytology. Although the follow-up with repeated testing was incomplete, it was very similar to that observed in the randomized controlled trials comparing HPV-based to cytology-based primary cervical screening (14). Nevertheless, a somewhat higher number of high-grade CIN would plausibly be detected with a more complete follow-up (6).

There is no internationally agreed standard reference HPV genotyping assay. The research-only Linear Array (LA) was among the assays most frequently used for genotyping in previous studies. However, LA test results are read manually and may be prone to interobserver variability. We genotyped the infections using the CE-IVD-marked CLART assay, which interprets the test results using a built-in software algorithm. In some studies (15–18) but not all (19), CLART and LA showed relatively good concordance. In our study of women undergoing primary screening, CLART detected a number of high-grade lesions similar to that of the HC2 and cobas assays. However, similar to cobas it did miss one of the three cases of cervical cancer (8).

### Comparison with other studies.

Taken together, the Horizon data are in good agreement with prior studies. Our summary of the literature comparing HPV detection by various assays found similarities between our SurePath-based findings and those from studies using other liquid media (5). More recently, Cook et al. compared HC2 and cobas on ThinPrep samples from the Canadian FOCAL randomized controlled trial of primary screening and observed a large difference in signal strength between samples where both assays tested positive (median RLU/CO, 54.9; median *C_T_*, 30.2) and samples where the assays returned different test results (HC2^+^/cobas^−^ median RLU/CO, 5.9; HC2^−^/cobas^+^ median *C_T_*, 38.3) (20).

Previous screening studies showed high levels of concordance between assays in terms of the detection of high-grade CIN, which corresponds to a substantially higher likelihood of detecting these lesions when multiple assays return a positive test result (Table 3). Nevertheless, it should be noted that only two studies reported CIN lesions detected jointly by cytology and two HPV assays, whereas the remaining studies reported CIN lesions detected only by cytology and one HPV assay. This potentially means that some of the variability in detecting CIN that was observed in our study, where all CIN were detected by cytology and/or up to four HPV assays, could not have been observed in previous studies. Several studies, including ours, however, reported relatively small numbers of high-grade CIN.

**TABLE 3:** cobas, CLART, and Aptima versus HC2: cross-sectional detection of ≥CIN2 or ≥CIN3 in primary screening at ≥30 years^*e*^

Study (country)	Compared assay	Storage medium	Age range (yr)	CIN grade	No. of detected CIN	Assay detecting CIN	HPV prevalence in CIN (no. positive/total no. [%])		No. detected			
							HC2	Assay	HC2^−^/assay^−^	HC2^+^/assay^+^	HC2^+^/assay^−^	HC2^−^/assay^+^
Horizon (DK)	Aptima^*a*^	SurePath	30–65	≥CIN3	38	Cytology, HC2, cobas, CLART, Aptima	35/38 (92.1)	34/38 (89.5)	2	33	2	1
Wu et al. (21) (CN)	Aptima^*b*^	STM^*c*^	25–59	≥CIN3	15	Cytology, HC2, Aptima	14/15 (93.3)	15/15 (100)	0	14	0	1
Nieves et al. (22) (MX)	Aptima^*d*^	ThinPrep	30–50	≥CIN3	16	Cytology, HC2, Aptima	16/16 (100)	16/16 (100)	0	16	0	0
Horizon (DK)	cobas	SurePath	30–65	≥CIN3	38	Cytology, HC2, cobas, CLART, Aptima	35/38 (92.1)	37/38 (97.4)	0	34	1	3
Heideman et al. (23) (NL)	cobas	UCM	29–60	≥CIN2	60	Cytology, HC2	55/60 (91.7)	54/60 (90.0)	5	54	1	0
Cook et al. (20) (CA)	cobas	ThinPrep	25–65	≥CIN2	94	Cytology, HC2	94/94 (100)	88/94 (93.6)	0	88	6	0
Lloveras et al. (24) (ES)	cobas	ThinPrep	23–63	≥CIN2	60	Cytology, HC2	59/60 (98.3)	59/60 (98.3)	0	58	1	1
Horizon (DK)	CLART	SurePath	30–65	≥CIN3	38	Cytology, HC2, cobas, CLART, Aptima	35/38 (92.1)	37/38 (97.4)	0	34	1	3

a Threshold cutoff for signal strength of ≥0.5 S/CO.

b Threshold cutoff for signal strength of ≥1.0 S/CO.

c ThinPrep for HC2.

d Threshold cut-off for signal strength not reported.

e Abbreviations: CA, Canada; CN, China; DK, Denmark; ES, Spain; HC2, Hybrid Capture 2; MX, Mexico; NL, The Netherlands; STM, sample transport medium; UCM, universal collection medium.

### Clinical implications.

The concordance between the assays increased with the severity of the underlying biology. The percentage of HPV-positive women testing positive on all four HPV assays increased from 22% in women with normal cytology to 68% in women with abnormal cytology and to 84% in women with ≥CIN2. The high level of concordance in detecting high-grade CIN indicates that the choice of an HPV assay for screening will have little effect on the high-risk women who should be recommended for treatment to avoid progression to cervical cancer. The likelihood that they will be detected through screening is high with any of the assays evaluated here, which is consistent with a relatively high clinical sensitivity for each assay.

On the other hand, false-positive screening tests, i.e., positive HPV test results with harmless infections that do not lead to high-grade CIN, represent clinically inconsequential noise. They represent a challenge for primary HPV-based screening even in women above 30 years of age (25), and this calls for further optimization of HPV assays. False positivity appears to be a hallmark of the weakly positive and discordant screening samples. Our data suggested that women with a single positive HPV test result are unlikely to harbor ≥CIN2, but they represented 40% of all HPV-positive women in our study, corresponding to ca. 10 to 20% of positive test results on each assay.

As their infections will be detected by some but not other assays, healthy women with false-positive test results will be affected by the choice of an HPV assay for primary screening. These women will be recommended for (unnecessary) follow-up, possibly including colposcopy. This brings into focus the question of whether the management recommendations for HPV-positive women should be the same irrespective of which assay detected the infection. Studies agree in that, regardless of the assay used for primary screening, HPV-positive women should not be directly referred for colposcopy (26–28). For HC2-positive women from the Dutch VUSA-screen study, Rijkaart and colleagues proposed using cytological triage and to repeat cytology testing at 12 months postbaseline for triage-negative women (26). This triage strategy had a negative predictive value for ≥CIN3 cases of >99% and required the lowest number of women referred for colposcopy. In this setup, the addition of HPV genotyping would lead to a higher cumulative number of colposcopies but would not significantly decrease the risk of missing ≥CIN3 cases. On the other hand, Iftner and colleagues, using data from the German Aptima- and HC2-based screening study (27), and Wright and colleagues, using data from the U.S.-based ATHENA study evaluating the cobas assay (28), found that optimal triage strategies seemed to involve HPV 16/18 genotyping at baseline. In these two studies, follow-up testing of triage-negative women could not be further evaluated, as immediate colposcopy was recommended to all HPV-positive women (for study purposes). Differences in study populations and designs may, to some extent, help explain the differences in the conclusions on the optimal management strategy for HPV-positive women. Another explanation is that the different selections of HPV-positive women chosen for follow-up by the different assays require adaptations in the clinical management. Given its relevance for policy-making in screening, this remains to be studied in more detail.

One of the indicators for monitoring the implementation of HPV vaccination is a change in the epidemiology of HPV genotypes (29). Our study suggested that none of the assays detects all HPV infections. The discordance between the assays in detecting the virus was observed also at the genotype level. For example, only 59% of all HPV 16 infections detected by either cobas or CLART were concordant on the two assays (data not reported). For HPV 18, this was 69%. Thus, to reliably attribute changes in HPV epidemiology to the use of the vaccine, it will be necessary to maintain consistency in the choice of the HPV assay.

In conclusion, discordance between multiple HPV assays in detecting HPV infections identified a cluster of weakly positive infections that are less frequently associated with HPV 16 and 12 other high-risk genotypes. As these samples also hardly ever harbored high-grade CIN, they were typically associated with false positivity in screening.

## Supplementary Material

Supplemental material
