# Supplementary material for: Differential Detection of Human Papillomavirus Genotypes and Cervical Intraepithelial Neoplasia by Four Commercial Assays
Source: J Clin Microbiol. 2016 Oct 24;54(11):2669–75. doi: 10.1128/JCM.01321-16 (PMC5078541; doi:10.1128/JCM.01321-16)
Supplement: Supplemental material [file supp_54_11_2669__index.html]

Supplemental material 

# Differential Detection of Human Papillomavirus Genotypes and Cervical Intraepithelial Neoplasia by Four Commercial Assays

## Supplemental material

- Supplemental file 1 -

  Assay testing protocols and Table S1 (Description of HPV infections in samples for which one, two, or three HPV assays returned a positive test result)

  PDF, 70K
