# Supplementary material for: Differential Detection of Human Papillomavirus Genotypes and Cervical Intraepithelial Neoplasia by Four Commercial Assays
Source: J Clin Microbiol. 2016 Oct 24;54(11):2669–75. doi: 10.1128/JCM.01321-16 (PMC5078541; doi:10.1128/JCM.01321-16)
Supplement: Supplemental material [file JCM.01321-16_zjm999095185so1.pdf]

University of Copenhagen
Department of Public Health

6 August 2016

Differential detection of Human Papillomavirus genotypes and cervical intraepithelial neoplasia by four commercial assays

SUPPLEMENTAL MATERIAL

Matejka Rebolj,[1#] Jesper Bonde,[2,3] Sarah Preisler,[2,3]
Ditte Ejegod,[3] Carsten Rygaard,[3] Elsebeth Lynge[1]

[1] Department of Public Health, University of Copenhagen, Copenhagen, Denmark
[2] Clinical Research Centre, Copenhagen University Hospital Hvidovre, Hvidovre, Denmark
[3] Department of Pathology, Copenhagen University Hospital Hvidovre, Hvidovre, Denmark

[#]Correspondence to: m.rebolj@qmul.ac.uk

HPV testing protocols in the Horizon study

Testing at the study baseline[1]

HC2

We used post-quot material that remained from the cytology procedure. As part of the cytology processing, post-quot material was diluted approximately 1:1 in SurePath. DNA was either denatured prior to testing by pre-treating manually in line with the manufacturer's CE-IVD protocol, or was isolated and purified using the DSP AXpH DNA kit on QIASymphony SP (QIAGEN, Hilden, Germany). Testing was undertaken on automated Rapid Capture System (RCS; QIAGEN, Gaithersburg, MD, USA). A minority of samples used for routine HC2 triage of women with ASCUS at age ≥30 years were denatured and tested manually.

Cobas

1 ml of the diluted material was aliquoted into a 13 ml round bottom test tube (Sarstedt, cat. no NC9018280) and stored at 2-8°C until testing. No pre-treatment of SurePath samples was required. Extraction of DNA was undertaken on cobas x480, and amplification and detection of high-risk HPV DNA on cobas z480 analyzer. Fluorescent TaqMan probes were used for detection of the amplicons during polymerase chain reaction (PCR) cycles. Amplification and detection of the 330-bp β-globin was used as an internal control of the testing processes.

[1] These testing protocols were published, with minor textual revisions, in Rebolj et al. PLOS ONE 2014;9:e86835.

CLART

1 ml of the diluted SurePath sample was spun down for five minutes at 14,000 revolutions per minute, with supernatant removed and cell pellet re-suspended in a mix of 180 µl phosphate buffered saline (10x conc. pH 7.4, Pharmacy product) and 20 µl Proteinase K (recombinant, PCR Grade, Roche Diagnostics, Rotkreuz, Switzerland). Samples were then vortexed and incubated for one hour at 56°C and one hour at 90°C. HPV DNA was purified using MagNa Pure LC 96 and MagNA Pure LC 32 instruments (Roche Diagnostics) with MagNA Pure LC Total Nucleic Acid Isolation Kit (Roche Diagnostics). PCR amplification was performed using the CLART HPV2 Amplification kit (Genomica). 5 µl of purified DNA were used for the PCR amplification. Prior to visualisation, the PCR products were denatured at 95°C for 10 minutes. Visualisation was performed using 10 µl of the denatured PCR products on the CLART microarray. Hybridisation between the amplicons and their specific probes on the microarray resulted in formation of an insoluble precipitate of peroxidase when adding a Streptavidin conjugate that binds to the biotin-labeled PCR products. The precipitate was analyzed automatically on the Clinical Array Reader (Genomica).

APTIMA

1 ml of the diluted sample was aliquoted into an APTIMA Specimen Transfer Tube containing 2.9 ml of buffered solution (Hologic/Gen-Probe). Samples were treated with proteinase K prior to testing, using the Pace 2 Fast Expression Kit containing 1 ml diluent and lyophilized reagent (all from Hologic/Gen-Probe). 100 µl of the reconstituted proteinase K was added to each Specimen

Transfer Tube and incubated at 65°C for two hours. The treated specimen tube was stored at 2-8°C until testing. Testing was performed on the PANTHER platform.

Follow-up testing for women with cytology-normal/HPV-positive test results at baseline

Testing on HC2 for all follow-up samples was performed on the post quot material with manual DNA denaturation followed by testing on the RCS.

Table S1. Description of HPV infections in samples with one, two or three HPV assays returning a positive test result. Comparison limited to detection of HPV by HC2, cobas or APTIMA.

		**HPV genotype distribution (row %)[a]**			
**HPV test results**	**N**	**HPV 16**	**Non-HPV 16 high-risk HPV**	**Only low-risk HPV infections**	**No HPV genotype**
**Primary screening at 30-65 years (N=2859)**					
1 positive	245	16 (6.5%)	69 (28.2%)	52 (21.2%)	108 (44.1%)
2 positive	100	26 (26.0%)	56 (56.0%)	9 (9.0%)	9 (9.0%)
3 positive	208	41 (19.7%)	147 (70.7%)	11 (5.3%)	9 (4.3%)
**Referral population (N=885)**					
1 positive	116	14 (12.1%)	33 (28.4%)	37 (31.9%)	32 (27.6%)
2 positive	79	13 (16.5%)	50 (63.3%)	13 (16.5%)	3 (3.8%)
3 positive	304	100 (32.9%)	190 (62.5%)	12 (3.9%)	2 (0.7%)

Abbreviations: HPV=Human Papillomavirus. Primary screening=women with a cytological sample at age 30-65 years with no recent cervical abnormalities (see Methods). Referral population=women with an abnormal cytological sample attending primary screening, or women attending follow-up for recent cervical abnormalities at any age (see Methods).

[a] As detected by the CLART assay.
